# Dying as a ‘normal’, ‘ordinary’ or ‘natural’ process in palliative care: a systematic scoping review and narrative synthesis

**DOI:** 10.1186/s12904-026-02195-w

**Published:** 2026-06-15

**Authors:** Hyun Woo Yu, Pretty Manyimo, John I. MacArtney

**Affiliations:** Warwick Medical School, University of Warwick, Gibbet Hill, Coventry, CV4 7AL UK

**Keywords:** Normal dying, Ordinary dying, Natural dying, Palliative care, Good death

## Abstract

**Background:**

The World Health Organization definition of palliative care describes dying as a ‘normal’ process and it, along with related ideas of ‘ordinary’ or ‘natural’ dying, is important in understanding what a ‘good’ death is. This scoping review aims to explore how dying has been described as ‘normal’, ‘ordinary’ or ‘natural’ in empirical palliative care literature to provide palliative care stakeholders with evidence-based insights into how this concept may enable quality dying experiences and a ‘good’ death.

**Methods:**

A scoping review was conducted by searching six online databases (Medline (via Ovid), Embase (via Ovid), PsycINFO (via Ovid), CINAHL (via EBSCO), Web of Science, and Scopus) for empirical English-language palliative care literature that explicitly described dying as ‘normal’, ‘ordinary’, ‘natural’, or their interchangeable terms ‘standard, ‘typical’ or ‘usual’. A narrative synthesis was used to present the clusters of features associated with their descriptions.

**Results:**

Twenty-one studies used ‘normal’, ‘ordinary’, ‘natural’ and ‘typical’ dying. They were often employed as a single statement despite some authors’ concerns regarding the lack of conceptual clarity. Interchangeability between ‘normal’ and ‘natural’ dying was observed. Understandings of ‘normal’, ‘natural’ and ‘typical’ were found to be diverse, subjective, and contextually bounded. Although there was some evidence that ‘normal’, ‘natural’ and ‘typical’ dying can influence the quality of dying experiences in palliative care, there was no evidence for ‘ordinary’ dying due to a lack of explicit use in the included studies.

**Conclusions:**

Understandings of dying as ‘normal’, ‘natural’ or ‘typical’ are held by clinicians and patients to play an important role in shaping the quality of dying experiences and providing a ‘good’ death in palliative care. However, the use of each idea remains heterogeneous, with conflicting definitions across terms. The diffuse evidence base suggests that the potential benefits, harms or limitations of their use in palliative care are therefore unclear. For this reason, we recommend caution regarding their use in practice or policy.

**Supplementary Information:**

The online version contains supplementary material available at 10.1186/s12904-026-02195-w.

## Background

Understanding what is ‘normal’ is of paramount importance in medicine [1, 2]. Ideas of what is ‘normal’ help define health and disease, shaping the goals of healthcare interventions and policies [1–3]. For the last 35 years, the World Health Organization (WHO) definition of palliative care has depended upon the understanding of dying as a “normal process” [4, 5]. However, what this affirmation actually means has neither been defined by the WHO [4, 5] nor been a matter of sustained or systematic research in palliative care [6–10]. This gap may stem from the seemingly commonsense impression that dying is simply a ‘normal’ process of life, making its empirical exploration appear redundant. Taking its dictionary definition (“ordinary or usual; the same as would be expected” [11]) and assuming the meaning is clear and shared appears to be sufficient, at least in everyday usage.

However, this reliance on common sense could be a problematic strategy for palliative care because the concept of ‘normal’ in healthcare has a complex biological and philosophical history. For instance, Boorse’s [12] Biostatistical Theory of Health defines ‘normal’ health as the biological functioning of the body’s parts and processes in line with species-typical norms. In this definition, each organ or system performs its role (e.g. the heart pumping blood, the lungs exchanging oxygen) at levels statistically expected within a reference class (e.g. a population of the same age, identifying gender). On the other hand, Sabshin [13] highlights the possible plurality in understanding what is ‘normal’ by identifying a four-fold framework – health (absence of disease), average (statistical mean), utopia (optimal functioning) and process (growth over time). Canguilhem [14], a philosopher of science, argued that ‘normal’ extends beyond a biological static average and is dynamically established through a person’s lived adaptation to changing conditions. This negotiation between descriptive conceptions (e.g. statistically ‘normal’ blood test results) and normative conceptions (e.g. ‘not normal’ results being redefined as ‘normal’ in chronic illness) underscores how understandings of ‘normal’ in healthcare are not uniform or fixed, but rather multifaceted and interrelated.

This is a salient issue in today’s palliative care because the concept of dying being a ‘normal’ – or its synonyms ‘ordinary’ or ‘natural’ – process is increasingly being used by palliative care leaders as a key element in improving the familiarity, confidence and openness needed for high-quality palliative care [15–20]. The opportunities to experience dying and discuss it are considered crucial in the effective development of death literacy, which remains challenging due to the prevailing perception of dying as a sensitive topic [21, 22]. In this regard, death literacy describes how “the behaviours, attitudes and beliefs of carers and their networks were transformed by the experience of caring, including: Seeing dying as a part of life; normalizing the experience of end of life caring at home and home death...” [23]^(p33)^.

Similarly, there have been efforts to establish an understanding of dying as an ‘ordinary’ process of life. Understood as such, dying should not be perceived as a medical process experienced only by a certain group of patients and healthcare professionals or a sensitive process of life that should be feared or treated as a taboo for discussion [24–26]. Instead, “ordinary dying” is a “forgotten wisdom” [27]^(p439)^ that should be reclaimed as a social process whereby a dying person addresses any unfinished agenda of their life, while their family and community take on the overall responsibility for their care [24–27]. It is argued that regaining this understanding would allow familiarity with dying to be restored, enabling more open discussion and education to occur, and subsequently fostering the knowledge, confidence, and openness needed for a good quality of dying [24–27].

However, those in palliative care professing the need to understand dying as ‘normal,’ ‘ordinary’ or ‘natural’ are often to be found asserting its benefits without citing research that validates the claims made [21, 22, 24–26]. This will be problematic to those who reason that medical practice should be based on peer-reviewed evidence. Indeed, studies have shown that the use of complex concepts without clear understanding can lead to suboptimal quality of care, or even patient harm [28–33]. Moreover, those with knowledge of medical social sciences and humanities will be acutely aware of how ‘normal,’ ‘ordinary’ or ‘natural’ are not merely “objective” clinical terms [34–39]. If we are witnessing a revival of their use, it will therefore be important to understand the relationships between the biological process alluded to and the normative contexts in which they are found, and how each is used to understand the other [40, 41].

To provide this we proposed to explore the evidence base for describing dying as a ‘normal’, ‘ordinary’ or ‘natural’ process in palliative care so that its benefits, harms and limitations could be better recognised in enabling quality dying experiences and a ‘good’ death. Based on our preliminary searches, there had been no synthesised literature serving this purpose of presenting the existing empirical evidence on the meanings and values associated with understanding dying as a ‘normal’, ‘ordinary’ or ‘natural’ process in palliative care. Therefore, this scoping review explored how dying has been described as a ‘normal’, ‘ordinary’ or ‘natural’ process in empirical palliative care literature, to provide palliative care stakeholders with useful insights into better recognising its meanings and values in enabling quality dying experiences and a ‘good’ death.

## Methods

### Design

The scoping review approach by Arksey and O’Malley (2005) [42] was used because of its usefulness in systematically synthesising the established evidence and current research gaps with regard to a topic of interest that has not been reviewed in the past [43]. This scoping review was conducted in line with the Preferred Reporting of Items for Systematic Reviews and Meta-Analyses extension for Scoping Reviews (PRISMA-ScR) [44]. The review protocol was prospectively registered with the Open Science Framework (Registration ID: osf.io/sy726).

### Eligibility criteria

In this study, the term ‘dying’ was defined as a lived, multidimensionally shaped experience of approaching the final event of life (death), extending beyond its biological understanding (the cessation of bodily functions) and not confined to the last hours or days of life [45, 46]. Based on the WHO definition and other literature, the term ‘palliative care’ was defined as: a healthcare and social care setting involving multidisciplinary professionals and a range of services (home, community, hospital, etc.) that provides a holistic environment for improving the quality of life of individuals and their families at any stage of the dying process, delivered through an approach focused on comfort and dignity [5, 47, 48].

A full list of inclusion and exclusion criteria can be found in Table 1. Studies were included if they were empirical research into palliative care that described the final process of life using the term ‘dying’ and as ‘normal’, ‘ordinary’, ‘natural’, or their three identical, interchangeable synonyms ‘standard’, ‘typical’ and ‘usual’ [49–51]. Studies were restricted to those published in English. No limits were set on research design, publication year, country of study, or the type of palliative care (e.g. primary or specialist palliative care, and adult or paediatric palliative care, etc).

**Table 1:** Inclusion and exclusion criteria

	Inclusion criteria	Exclusion criteria
Population	Literature on dying of humans	Literature on dying of: -fictional figures -robotics -animals -plants -microorganisms e.g. bacteria, fungi -human body organisations e.g. cells, tissues, organs
Concept	Explicit descriptions using the term ‘dying’ and as ‘normal’, ‘ordinary’, ‘natural’, ‘typical’, ‘usual’ or ‘standard’	Implicit descriptions of dying e.g. using the terms ‘ageing’, ‘living’, ‘death’
Context	Palliative care	Not palliative care e.g. post-mortem
Quality of evidence	Peer-reviewed literature	Non-peer reviewed literature -conference abstracts -editorials -opinions -guidelines -policies -books/book chapters -magazine articles -blogs -documentary films -theses/dissertations
Types of evidence	Qualitative or Quantitative Empirical research into palliative care.	Fictional or non-empirical based research or reflections on dying or palliative care e.g. from drama, literature studies, abstract philosophy etc.
Source of evidence	Medline (via Ovid) Embase (via Ovid) PsycINFO (via Ovid) CINAHL (via EBSCO) Web of Science Scopus	Not available in the chosen databases Not available via the Warwick University access Monographs, including (auto)biographies.
Publication year	No limit	-
Language	English	Non-English: this includes literature that only its abstract is published in English
Country of study	No limit	-

### Information sources and search strategies

Six online databases were searched: Medline (via Ovid), Embase (via Ovid), PsycINFO (via Ovid), CINAHL (via EBSCO), Web of Science, and Scopus. Along with palliative care search filters [43], the search terms used were: (“dying” AND (“normal” OR “ordinary” OR “natural” OR “usual” OR “standard” OR “typical”)) (please refer to Supplementary Document 1.1 in Additional file 1 for details).

Studies that contained a description of dying as ‘normal’, ‘ordinary’, ‘natural’, ‘standard’, ‘typical’ or ‘usual’ within their title or abstract were included. The reference lists of all literature eligible for final inclusion were manually screened to identify any additional studies that met the inclusion criteria.

### Study selection and data extraction

Study selection was conducted using the Rayyan software application. Studies were screened independently by two reviewers (HY and PM). Titles and abstracts were evaluated in line with the inclusion and exclusion criteria shown in Table 1. Full texts were retrieved and screened when the relevance of a study was not clear from the abstract. Any disagreements between the two reviewers regarding study inclusion were discussed and a consensus was reached. Any studies where the two reviewers could not reach an agreement were referred to the third reviewer (JM) for the final assessment. The remaining studies formed the final list of literature included for analysis. From this final set of literature, relevant data were charted using a data extraction table (please refer to Supplementary Table 1 in Additional file 1 for details).

### Quality appraisal

Quality appraisal of included studies is not mandatory in a scoping review and depends upon its purpose [42]. A quality appraisal was not conducted here because this review did not aim to assess the efficacy or effectiveness of using the terms of interest in palliative care. However, the narrative synthesis of the results included a critical appraisal of the occurrence and meaning of the terms.

### Synthesis of results: narrative synthesis

A narrative synthesis approach, modified from the framework by Popay et al. (2006) [52], was used, which is a suitable analytic approach when dealing with heterogeneous or limited data, particularly where there is a need for conceptual exploration. First, all explicit descriptions of ‘normal’, ‘ordinary’, ‘natural’, ‘standard’, ‘typical’ and ‘usual’ dying in the included studies were summarised according to their individual categories (e.g. grouping all narratives describing dying as ‘normal’ under the heading ‘normal dying’): these data have been provided as a supplementary document (please refer to Supplementary Table 2 in Additional file 2 for details). The descriptive data were interpreted iteratively to develop clusters of features representing ‘normal’, ‘ordinary’, ‘natural’, ‘standard’, ‘typical’ and ‘usual’ dying in palliative care.

## Results

### Search results

**Fig. 1:**
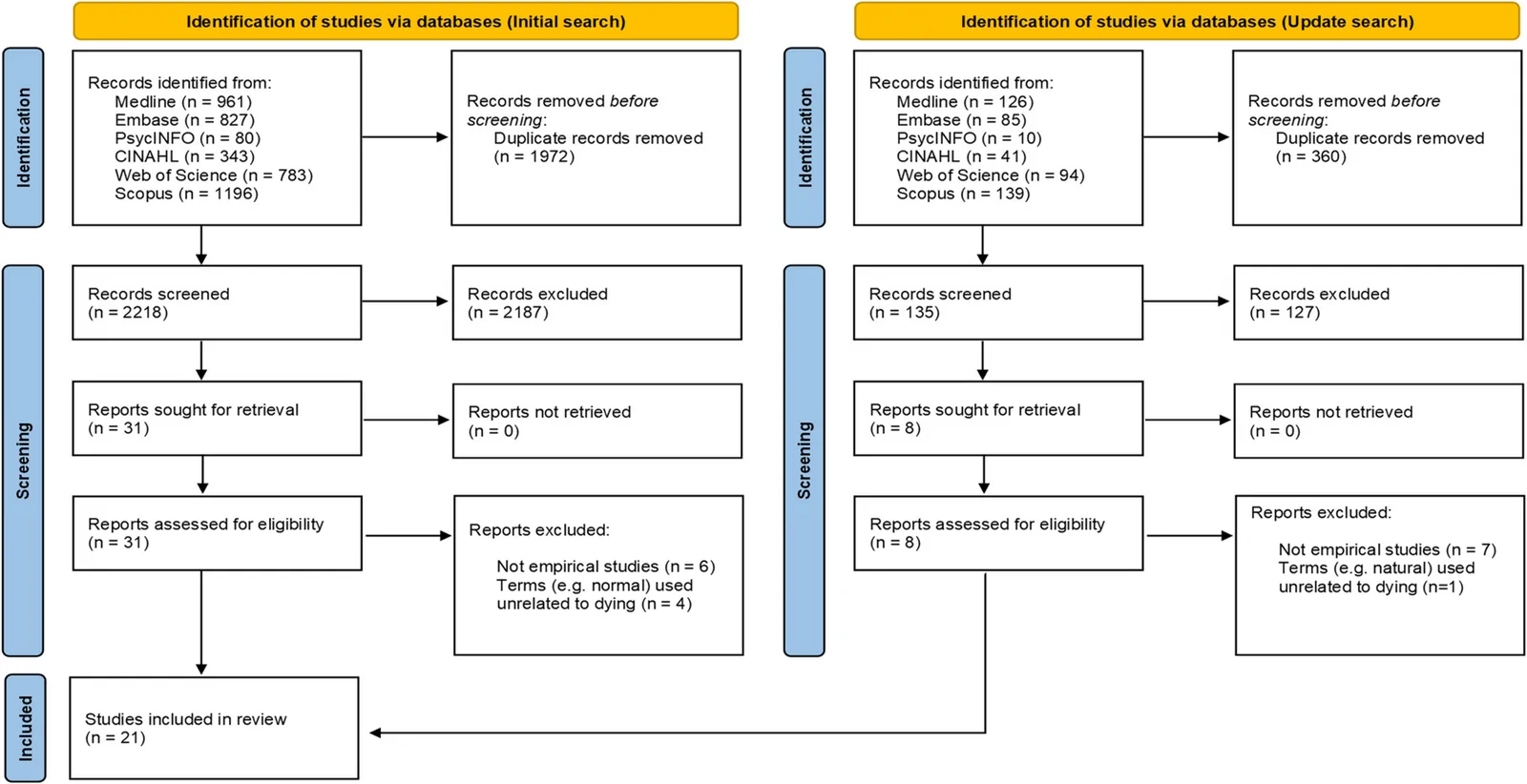
PRISMA flow diagram

An initial database search was conducted in December 2023. After duplicates were removed, 2,218 studies were screened for eligibility, among which 21 studies were included for analysis. An update search was undertaken in November 2025, and no new studies were found that met the eligibility criteria. The selection process is presented using the PRISMA flow diagram [53] below (Fig. 1):

The summarised findings have been presented in Supplementary Table 2 in Additional file 2.

### Study characteristics

The publication years ranged from 2007 to 2023. Studies were conducted in the US (*n* = 7) [54–60], Australia (*n* = 2) [61, 62], Nigeria (*n* = 2) [63, 64], Switzerland (*n* = 2) [65, 66], Canada (*n* = 1) [67], Finland (*n* = 1) [68], Germany (*n* = 1) [69], Indonesia (*n* = 1) [70], Japan (*n* = 1) [71], Norway (*n* = 1) [72], Sweden (*n* = 1) [73], and the UK (*n* = 1) [74].

Twelve studies [57–60, 65, 66, 68, 69, 72, 73] were qualitative (interviews (*n* = 8) [57–59, 65, 68, 69, 72, 73], participant observation and interviews (*n* = 1) [67], case study (*n* = 1) [60], a combination of case study, focus group interviews and single interview (*n* = 1) [66], and a combination of focus groups and interviews (*n* = 1) [56]), eight studies [55, 61–64, 70, 71, 74] were quantitative (cross-sectional surveys (*n* = 6) [61–64, 70, 71], longitudinal surveys (*n* = 1) [74], and pre-test and post-test surveys (*n* = 1) [55]), and one study [54] used mixed methods (cross-sectional surveys and interviews).

Five studies [58, 62–64, 71] were in hospital settings, four studies [59, 60, 67, 69] in hospice, four studies [65, 66, 68, 72] in multi-sites (home and hospice (*n* = 1) [68], home and hospital (*n* = 1) [66], hospital and kindergarten (*n* = 1) [72], and hospice, hospital and nursing home (*n* = 1) [65]), three studies [55, 70, 73] in universities, two studies [54, 56] in ethnic-specific communities, one study [57] in home, one study [74] in GP surgeries, and one study [61] in adult day care centres.

In terms of participants, six studies [58, 59, 62–64, 71] were with healthcare professionals only; three studies [57, 68, 74] with patients only; three studies [55, 70, 73] with clinical students; three studies [54, 56, 61] with community members who are not patients; two studies [65, 66] with a mixture of patients, relatives and healthcare professionals; two studies [67, 69] with a mixture of healthcare professionals and voluntary workers; one study [72] with a mixture of healthcare and non-healthcare professionals; and, one study [60] with patients and relatives.

Participants (Korean American older adults) in Hong et al. [56] used the term ‘natural’ explicitly in its grammatical variation ‘naturally’ (“I want to die naturally…” [56]^(pE215)^). In the other 20 studies [54, 55, 57–74] the terms of our interest were not used by participants in describing dying i.e. they were only used by the study authors.

Authors used the following terms of our interest explicitly: in eight studies [56, 57, 59, 68, 69, 71–73] the authors used the term ‘natural’; in seven studies [54, 60–64, 70] the authors used ‘normal’; in two studies [65, 74] authors used ‘typical’; and, in four studies [55, 58, 66, 67] authors used both ‘natural’ and ‘normal’. The term ‘ordinary’ was not used by any of the included studies, although the authors of one study [68] used its grammatical variation ‘ordinariness’ once in its abstract (“Although the ordinariness of dying in hospice settings was sometimes too much to bear…” [68]^(p720)^).

When authors used the term(s) of our interest, seven studies [55, 58, 64, 65, 70, 72, 74] did so in the introduction-background section; four studies [54, 61, 62, 71] in the methods section; 14 studies [54–56, 59, 61–64, 66–69, 71, 74] in the results section; 10 studies [58, 59, 61–64, 66, 67, 71, 74] in the discussion/commentary section; one study [71] in the conclusion section; and, three studies [57, 60, 73] in the abstract only.

### Narrative synthesis

#### Single-statement descriptions of ‘normal’, ‘ordinary’, ‘natural’ and ‘typical’ dying

This review found that the included studies explicitly used the terms ‘normal’ [54, 55, 58, 60–64, 66, 67, 70], ‘ordinary’ [68], ‘natural’ [55–59, 66–69, 71–73] and ‘typical’ [65, 74] (and/or their grammatical variations), but not ‘standard’ or ‘usual’, to describe dying within palliative care settings. Both the authors and the participants in all but one study appeared to describe dying as ‘normal’ [54, 55, 58, 60–64, 70], ‘ordinary’ [68], ‘natural’ [55–59, 66, 68–70, 72, 73] or ‘typical’ [65, 74] in the form of a plain statement that did not necessitate further definition (e.g. the authors of Colcough and Brown [54] used the “dying is a normal part of life” statement in their survey without clarification). The authors of Wright et al. [67] were the only researchers to provide an explicit definition. They did so when describing how hospice caregivers explained to families about dying using both ‘normal’ and ‘natural’:

With this text, families were introduced to the various bodily changes in dying as the “normal, natural way in which the body prepares itself to stop” [67]^(p962)^.

Yet as this example shows, the amount of detail in the definition was succinct. This suggested that – regardless of whether a study provided a definition for ‘normal’, ‘ordinary’, ‘natural’ or ‘typical’ dying – understandings of those terms were treated as self-evident, requiring little explanation beyond what is commonly understood as such.

In studies [54, 56, 57, 59–64, 68–73] where authors only used either ‘normal’ or ‘natural,’ but not both, their choice appeared to be in line with the dictionary definitions: of ‘normal’ as “ordinary or usual; the same as would be expected” [11]; and, of ‘natural’ as “found in nature and not involving anything made or done by people” [75]. ‘Natural’ seemed to have been used when the involvement of human intervention (regardless of its involvement being expected) was more the issue at stake [56]; whilst ‘normal’ was used in relation to the expectedness of the process (regardless of involvement of human intervention) [60].

#### Interchangeability between ‘normal’ and ‘natural’ dying

We found there was some interchangeability of ‘normal’ and ‘natural’ in describing dying, without considered reflection on the (potentially contradictory) differences in meaning. Zulfatul A’la et al. [70] used ‘normal’ and ‘natural’ without drawing any distinction, saying in the Abstract, “Dying is a normal human phenomenon…” [70]^(p25)^ and in the Background section, “Death is a natural human phenomenon” [70]^(p25)^. Similarly, Wright et al. [67] appeared to use both ‘normal’ and ‘natural’ as complementary terms in describing the bodily changes during dying.

No similar interchangeability was observed between ‘ordinary’ and ‘typical’, in relation to each other or to ‘normal’ and ‘natural’. However, the meaning of this is difficult to ascertain due to their limited occurrence in the included studies.

#### Diversity, subjectivity and contextuality of ‘normal’, ‘natural’ and ‘typical’ dying

Included studies suggested that diverse views could exist on what is ‘normal’ about dying. In those quantitative studies that explored the affirmation that ‘normal’ dying is part of palliative care, the majority of the participants stated agreement with this view: of the 92 participants 76% (n = not given) answered ‘Strongly agree’ and 14% (n = not given) ‘Agree’ for this affirmation [54]; of 171 participants 92.64% (*n* = 151) answered either ‘Strongly agree’ or ‘Agree’ [61]; of 82 participants 80.5% (*n* = 66) answered ‘Yes’ to this affirmation [63]; and, of 148 participants 78.4% (*n* = 116) answered ‘Yes’ [64]. However, enough participants in those studies disagreed with this view, or were neutral or unsure about it, to imply that this is not a universally accepted view: of 92 participants 4% (n = not given) answered ‘Neither agree nor disagree’ for this affirmation [54]; of 171 participants 3.68% (n = 6) answered ‘Neutral’ and 3.68% (n = 6) either ‘Disagree’ or ‘Strongly disagree’ [61]; of 82 participants 17.1% (n = 14) answered ‘No’ to this affirmation and 2.4% (n = 2) ‘Don’t know’ [63]; and, of 148 participants 15.9% (n = 23) answered ‘No’ and 4.8% (n = 7) ‘Don’t know’ [64]. Some studies [61, 63, 64] suggested that the diverse views on this matter could be due to inadequate understanding of what palliative care was. For example, Fadare et al. (B) [64] attributed this diversity to “availability or lack of training opportunities in palliative care at both undergraduate and postgraduate levels” [64]^(p4)^.

Included studies also showed how understandings of ‘normal’, ‘natural’ and ‘typical’ dying could be subjective. For instance, the authors of Shepherd et al. [62] found that dying in one setting (e.g. home) was perceived by their participants as “a more normal process” [62]^(p4)^, in contrast to dying in other settings such as the emergency department or a residential aged care facility. Similarly, Saladin et al. [66] observed that Voluntarily Stopping of Eating and Drinking could be perceived as both ‘natural’ and ‘unnatural’ dying depending on the contexts (as presented in Section 3.3.5) in which it was understood and experienced. Gott et al. [74] found there was a discrepancy between theoretical ‘typical’ dying in heart failure and that in reality. The large variations in deterioration observed in this study [74] suggested that either ‘typical’ dying in this patient group may not exist or the trajectories are so individualised that “there is a need to reconsider current efforts to plan and deliver services in heart failure on the basis of the theorised trajectory until further evidence is available” [74]^(p98)^.

Two studies [59, 71] highlighted the inadequacy within their design of having statements about ‘natural’ dying, without clarification as to what that is:

Three participants had concerns about the wording of the definition, including terms such as ‘‘disruption’’ or ‘‘natural dying process,’’ which may be too vague to inform safety metrics or policies [59]^(p542)^.

… some limitations need to be considered… Fifth, the term “Natural dying process” was not defined and depended on respondents’ interpretation [71]^(p116)^.

Due to a lack of descriptions of dying as ‘ordinary’ in the included studies, it was not possible to ascertain whether similar diversity and subjectivity were present.

#### Synonyms and antonyms of ‘normal’, ‘natural’ and ‘typical’ dying

Synonyms refer to words with identical or closely related meanings [76]. Four synonyms were identified, based on using them to refer to, or in place of, ‘normal’, ‘natural’ and ‘typical’ dying: “Letting go” [67]^(p961)^ for ‘normal’; “Voluntarily Stopping of Eating and Drinking” [66]^(pp1, 3, 8–10)^ for ‘natural’; “theoretical dying trajectories” [74]^(p98)^ for ‘typical’; and, “life’s fundamental realities” [58]^(p433)^ for both ‘normal’ and ‘natural’.

Antonyms refer to words that have opposite or contrasting meanings [77]. Four antonyms were identified based on their usage to refer to a process in opposition to ‘natural’ dying: “Voluntarily Stopping of Eating and Drinking” [66]^(pp1, 8, 10)^; “poison/poisoning [oneself]” [66]^(p10)^; “killing [oneself]” [66]^(p10)^; and, “assisted suicide/suicide” [66]^(pp1, 3, 8–10)^.

In Saladin et al. [66] Voluntarily Stopping of Eating and Drinking was used both as a synonym and an antonym of ‘natural’ dying. As presented in Sections 3.3.3 and 3.3.5, its authors explained that this was due to the context-dependent nature of how it was understood [66].

#### Factors identified in the studies as influencing the understanding of ‘normal’, ‘ordinary’ and ‘natural’ dying

Saladin et al. [66] noted that both the age at which dying is experienced, and the age of the person who perceives dying, influenced how Voluntarily Stopping of Eating and Drinking was situated ‘in the field of tension between suicide and natural dying’ [66]^(p9)^. Some participants (registered nurses and relatives) in this study viewed that Voluntarily Stopping of Eating and Drinking in older age groups (> 76 years old) is ‘classified as natural dying’ [66]^(p8)^ but as suicide in younger age groups (< 65 years old) [66]. However, this perception of a clear ‘natural’/suicide (or ‘not natural’) binary became obscure in the in-between age group (between 65 and 76 years old), where ‘a conflict happened in the given situation’ [66]^(p8)^ regarding the ‘naturalness’ of Voluntarily Stopping of Eating and Drinking.

Some of the included studies [56, 66, 73] stated that both theoretical and experiential knowledge of dying were important for understanding dying as a ‘natural’ process. How dying is communicated among clinicians, patients and carers was also observed by some of the authors as important for understandings of ‘normal’ [67] and ‘natural’ [66, 67] dying. However, some authors raised concerns about inadequate exposure and training for acquiring the knowledge and communication skills on ‘normal’ [63, 64, 67] and ‘natural’ [55, 67] dying. Saladin et al. [66] and Wright et al. [67] mentioned the consequential lack of confidence and familiarity in the care of the dying when clinicians and family did not have an understanding of dying as ‘normal’ [67] or ‘natural’ [66, 67].

Culture, religion and faith were also identified as factors affecting the use and meaning of ‘normal’ and ‘natural’. Saladin et al. [66] and Wright et al. [67] observed that their participants established their ‘normal’ [67] and ‘natural’ [66, 67] dying understanding from socio-institutional cultures such as those of hospice and hospital in which patients were cared for. Saladin et al. [66] and Viitala et al. [68] observed how some of their participants viewed that faith and religion could play a positive role in understanding and accepting (or not) dying as a ‘natural’ process [66, 68], or in recognising its ‘ordinariness’ [68].

#### Impacts of ‘normal’, ‘natural’ and ‘typical’ dying on experiences of palliative care and dying

Levy et al. [60] and Shepherd et al. [62] discussed the effects of ‘normal’ dying on experiences of dying symptoms and upon place of dying decision-making. Levy et al. [60] observed that perceiving “End-of-Life Dreams and Visions” [60]^(p1549)^ as a ‘normal’ dying symptom can help strengthen the relationship between the child and her mother and alleviating the fears around death. Shepherd et al. [62] found that dying being ‘normal’ was an important factor for participants in deciding preferred place of dying, for which home and hospice/palliative care unit were preferred to hospital and residential care facilities.

The ways that ‘normal’ and ‘natural’ dying could be invoked to manage the emotional aspects of dying was also evident, especially in relation to clinical (shared) decision making, medico-legal issues, and clinical practice guidelines. Österlind et al. [73] discussed how using ‘natural’ dying increased the likelihood of dying being perceived as something “good” [73]^(p14)^, “beautiful” [73]^(p14)^, “peaceful” [73]^(p14)^ or “nice” [73]^(p14)^, rather than something “frightening” [73]^(p14)^. Hogstad et al. [72] observed that understanding dying as a ‘natural’ process helped clinicians frame decision-making about treatments and ongoing care. Similar usage was notable in descriptions of what was ‘natural’ about Voluntarily Stopping of Eating and Drinking decisions, as well as what treatments and medications are acceptable to give [56] (or not to give e.g. antipsychotics for hypoactive delirium [71], or to stop [69]) during dying and when the care approach should move from curative to palliative [58]. Wright et al. [67] also discussed how an understanding of ‘normal’ or ‘natural’ dying can improve families’ familiarity with the dying process, with the hope that it would provide good death experiences. Understanding dying as ‘typical’ was also used as a theoretical framework for developing service planning, but when individual dying process was too diverse it could lead to inappropriate use of resources that only benefitted a small patient group [74]. Similarly, Ohnsorge et al. [65] stated that as ‘typical’ dying in organ failure is unpredictable, clinicians often find it difficult to have open discussions with patients about dying and related issues due to associated prognostic uncertainty, resulting in delayed and reduced access to palliative care.

Yet, some of the included studies highlighted that the impacts of ‘normal’ dying on experiences of palliative care and dying can be limited. For instance, as described in the preceding paragraph, the authors of Österlind et al. [73] observed a shift in the emotional connotations of dying (e.g. from something “frightening” [73]^(p14)^ to something “nice” [73]^(p14)^) when it was perceived as ‘natural’. In contrast, the authors of Zulfatul A’la et al. [70] stated that dying remains as “a painful experience for human beings and their family” [70]^(p26)^ despite it being a ‘normal’ process. Similarly, the authors of Ohr et al. [61] observed contradictory attitudes toward dying and death, noting that “whilst there was an acceptance of dying as a normal part of life, almost half of the respondents (n = 79) believed that death should be avoided at all costs” [61]^(p1684)^. The authors attributed this paradox – accepting a process (dying) as ‘normal’ but rejecting its end event (death) as such – to “the culture of ‘do everything’” [61]^(p1686)^, suggesting the potential limitation of a ‘normal’ dying understanding in shaping the quality of dying (and death) experiences when other perspectives, such as cultural understandings, are present.

## Discussion

### Inadequate clarity in understandings of ‘normal’, ‘ordinary’, ‘natural’ and ‘typical’ dying

This review found that empirical palliative care literature employed explicit descriptions of dying as ‘normal’ [54, 55, 58, 60–64, 66, 67, 70], ‘ordinary’ [68], ‘natural’ [55–59, 66–69, 71–73] and ‘typical’ [65, 74] (and/or their grammatical variations) within palliative care settings. However, they were often used in the form of plain statements without further elaboration on their meanings. Our analysis also showed a diversity in the use and understanding of these terms, implying the presumptive use of (potentially) complex concepts merely in the form of a statement without formal validation of what they mean. This paucity of definition or contextualisation appeared at odds with the concerns raised by some of the included studies about the lack of clarity surrounding these concepts [59, 61].

This is problematic in many respects. Clinically, it could undermine the reliability of practice derived from unproven concepts of ‘normal’, ‘ordinary’ and ‘natural’ (and ‘typical’) dying, increasing the risk of ineffective or even harmful patient outcomes that could compromise patient-clinician trust and patient safety [78]. Sociologically, it could destabilise public trust in palliative care, exacerbate health inequities by exposing vulnerable populations to unproven practices, and weaken the legitimacy of palliative care as a socially accountable practice [79, 80]. Ethically, palliative care professionals may inadvertently mislead patients or compromise informed consent when relying on concepts without substantiating their meaning, efficacy or usefulness [78].

Therefore, it is pertinent to address this lack of empirical evidence, so that the utility of ‘normal’, ‘ordinary,’ ‘natural’ and ‘typical’ dying concepts could be formally examined and validated. Further research could consider exploring the lived views of diverse palliative care patients, clinicians and policy makers on the meanings and values of ‘normal’, ‘ordinary,’ ‘natural’ and ‘typical’ dying, to build a stronger empirical framework upon which each group could base their understanding regarding this matter. In particular, this should include the lived views of patients and their families, considering the paramount importance of patient-centredness in palliative care [81].

### The heterogeneity of ‘normal’ dying

This review found that dying being a ‘normal’ process is not a universally held view in palliative care. There were clinicians, patients and families who strongly believed that dying is a ‘normal’ process [54, 61, 63, 64], while there were others who questioned the empirical (physiological) and normative basis on which any ‘normality’ could be established. Included studies attributed this heterogeneity to a lack of good understanding of what palliative care is [63, 64] and a lack of clarity about what ‘normal’ dying means [61], both of which, according to the wider literature [11, 82, 83], may stem from the increasing complexity of caring for the dying. Indeed, an ageing population with associated multiple co-morbidities and advancements in healthcare interventions has made dying trajectories more complex and less predictable, which may have consequently made the ‘normalcy’ (in terms of its expectedness) of dying become consequently more obscure [11, 82, 83].

The heterogenous understanding of ‘normal’ dying could also be due to the context-dependent nature of its understanding as found in this review, which aligns with findings elsewhere regarding the importance of context for conceptualising particular terms within healthcare settings [84–86]. For example, we found that understanding dying as a ‘normal’ process influenced decision-making on the place of dying (e.g. home, hospice) [62]. However, caution is needed when associating the aspiration to be cared for and die in particular locations as part of ‘normal’ dying. While there is a distinct policy push for greater numbers of people to be cared for and die in community settings [87], it is not evident that this is something most people want [88], nor is it an option that is equally available to all [89, 90]. Indeed, as people come to the last days and weeks of life the wish to be cared for and die in an institutional setting could be explainable as the more ‘normal’ desire and situation to find oneself in at the end of life, especially in countries with greater availability of specialist palliative care [91, 92]. It is therefore pertinent to explore which locales of (specialist) palliative care the understanding of ‘normal’ dying are entangled with, as well as the potential benefits, harms and limitations of this understanding within clinical decision-making processes.

More broadly, future research may need to examine the shifting bio-social status of asserting dying as a normal process that has gained medicine’s attention vis-à-vis a pathological status that was previously notable for being mostly neglected by medicine [14, 93]. The literature we have explored suggests that the transformation in both the moral economy of achieving a ‘normal’ death and the bioscientific problematisation of how dying can be understood as a ‘normal’ process are interrelated in, as yet, unexplained ways.

### ‘Natural’ dying is not a neutral description

Understanding dying as a ‘natural’ process was found to be ambiguous due to its context-dependent nature, in that contextual factors may not always provide a clear understanding of what is and is not ‘natural’ in dying. As seen in the case of age and Voluntarily Stopping of Eating and Drinking, ‘natural’ and ‘unnatural’ dying may not exist as separate concepts but are related as part of a conceptual spectrum [66]. That study [66] also noted that Voluntarily Stopping of Eating and Drinking in one context (> 76 years old) was part of ‘natural’ dying, but was perceived as ‘unnatural’ in another (< 65 years old), and a superposition of ‘natural’ and ‘unnatural’ in the in-between age group (between 65 and 76 years old). Therefore, blurred boundaries between what is ‘natural’ and ‘unnatural’ about dying, and the associated ambiguity in the understanding of ‘natural’ dying in palliative care, may not be an unexpected challenge surrounding this matter.

Added to this, there is a degree of deviation from a ‘commonsense’ dictionary definition of ‘natural’ (i.e. “not involving anything made or done by people” [75]) that is considered acceptable in what counts as ‘natural’ in healthcare [94–96]. For example, the use of synthetic opioid painkillers such as buprenorphine may be regarded as part of providing a ‘natural’ death [97, 98]. What may add further complexity to understanding ‘natural’ is that the extent to which ‘unnatural’ is acceptable in ‘natural’ (for it to remain ‘natural’) is not fixed but clinically and socio-culturally negotiated. For instance, interventions such as epidurals or inductions may be deemed acceptable in ‘natural’ childbirth, clinically if they are perceived as supportive rather than disruptive to its ‘natural’ process, and socio-culturally and ethically depending on various factors such as traditional norms, maternal autonomy (mother’s choice versus institutional protocols), and safety [99–101].

This alludes to the likely dynamic and normative nature of understanding what is ‘natural’ in healthcare, which needs to be continually shaped, de-shaped and re-shaped as clinical, socio-cultural and ethical factors evolve and intertwine [34–36, 41]. The same could be the case for the understanding of ‘natural’ dying amid evolving palliative care settings, since traditionally held views on ‘natural’ dying (or views held as commonsense) may lose their meanings and values in changing contexts. For instance, Walter [34] cautions against romanticised assumptions that traditional societies experienced death more ‘naturally’ or nobly, arguing that such views oversimplify the diverse cultural meanings of dying, death and bereavement and risk imposing idealised meanings and values onto their contemporary experiences. In this regard, what is concerning is the paucity of socially and historically informed empirical palliative care research on ‘natural’ dying.

That is not to say that we did not find evidence of the ambiguity in understanding what is ‘natural’ (and ‘unnatural’) about dying in empirical palliative care literature [66]. However, despite the fact that it has been more than 30 years since the above-mentioned cautions were advised by Walter [34], empirical inquiry on this matter, including any attempt to elucidate this ambiguity, appears to have stopped there. While studies [102, 103] have explored other normatively constructed palliative care concepts such as ‘good death’ and caregiver experiences, there appeared to be a notable lack of research specifically addressing ‘natural’ dying in palliative care. Consequently, it is difficult to ascertain the current state of how ‘natural’ dying is being understood and used in palliative care, specifically whether clinicians, patients and policy makers have been uncritically adopting an oversimplified (dictionary) understanding, and if so, whether its limitations or even potential harms are being overlooked. Further research could therefore explore the forms of ambivalence in understandings of ‘natural’ dying through discussion among palliative care clinicians, patients and policy makers about the range of ‘natural’ (and ‘unnatural’) dying present in diverse palliative care contexts.

The ideas of dying being a ‘natural’ process were also found to influence the decision-making process in palliative care, particularly on the acceptability of treatments and the stage of dying at which they are considered appropriate. This aligns with how the understanding of ‘natural’ in wider healthcare settings has been used, often in opposition to artificiality (or ‘unnatural’), to define what is morally good, right or acceptable [41, 94, 96, 104]. However, similar to ‘normal’ dying, the wider literature suggests that the effects of this influence are likely to be context-dependent rather than the normative weight of ‘natural’ dying being inherently good or bad. For instance, Inouye et al. [105] found a negative correlation between understanding hypoactive delirium as a ‘natural’ part of dying and the use of antipsychotics, highlighting a potential benefit of the ‘natural’ dying concept in reducing the use of medications with harmful side effects during dying, which is considered inappropriate. However, the same ideas of ‘natural’ dying could discourage the use of opioids (human-made painkillers that can pose harmful side effects like antipsychotics) for moderate-to-severe pain in people dying with advanced cancer, a recommended practice in palliative care [106, 107]. The reasons why the ideas of ‘natural’ dying have such a range of influences in palliative care, and whether these are something that need to be enabled, maintained or abandoned, require further exploration before any strong recommendations can be made for their use in clinical practice or palliative care policy.

### The value-ladenness of ‘typical’ dying

We found that understandings of ‘typical’ dying were suggested as playing an important role in improving prognostication at the end of life, by providing a theoretical framework upon which prognostic tools can be developed [65, 74]. Included studies [65, 74] speculated that if dying were better typified by its common patterns, characteristics and trajectories, clinicians’ ability to identify when a patient is approaching the end of life might be improved, which could in turn enhance the confidence in initiating conversations about unmet palliative care needs with patients. Included studies [65, 74] argued that, by doing so, care could be more precisely aligned with individual patient preferences and be provided in a more timely manner, improving the overall experience of dying. This is supported by the wider literature [108–110] arguing that prognostic accuracy is important for effective communication on dying, timely access to palliative care services and patient-centred care planning. However, prognostic accuracy remains a significant challenge in palliative care [111–117], and this is not only in organ failure – as found in this review – but also in other terminal illnesses [110, 118, 119]. A better understanding of how ideas of ‘typical’ dying are used in these contexts may therefore provide important insights into how prognostic accuracy is communicated and ultimately the quality of palliative care experiences.

Yet, in this regard, it seems striking that despite the vast body of work on prognostication in palliative care, the use of ‘typical’ dying appears to be limited in this context [120–124]. This could be because the term may not have yet gained traction as a meaningful analytic category within palliative care research, similar to what we found regarding the limited usage of its synonyms ‘normal’, ‘ordinary’ and ‘natural’ dying. Alternatively, as one of our included studies [74] observed, it could be because dying trajectories are so diverse that ‘typifying’ dying into a classifiable process is either impossible if meaningful patterns do not exist in reality to begin with, or futile if it cannot provide useful information for individuals experiencing dying.

The latter is an especially salient issue that warrants critical scrutiny through further research because, similar to ‘normal’ and ‘natural’ dying, ‘typical’ dying should also be expected to be value-laden. Hence, its mis-affirmation could result in harms to palliative care by oversimplifying individual, unique dying experiences. For instance, in defining health Boorse’s Biostatistical Theory states that “a normal function of a part or process within members of the reference class is a statistically typical contribution by it to their individual survival and reproduction” [12]^(p555)^. What Kingma [125] critiques about such understanding of ‘typical’ is that it undermines its value-ladenness, in that typical functioning is shaped by normative judgements and not just what are taken as empirical facts. This implies that while statistical and epidemiological rationales may justify the construction of ‘typical’ dying trajectories, such ideal types may not correspond to lived realities. This leaves the salient question regarding for whom the dying is ‘typical’ left unaddressed [126, 127]. An uncritical acceptance of ‘typical’ dying or a presumptive attempt to ‘typify’ dying in palliative care could hence risk flattening the richness of individual, unique experiences of dying into a generalised abstraction, inadvertently obscuring opportunities for patient-centred care by overlooking the nuances of personal, cultural, and relational contexts.

### The discrepancy between colloquial and evidence-based usage of ‘ordinary’ dying

None of the included studies explicitly used the term ‘ordinary’ to describe dying, although the idea of dying as an ‘ordinary’ process was expressed in one study [68] through its grammatical variation ‘ordinariness’. The complete absence of the explicit use of the term ‘ordinary dying’ in the palliative care research literature was nevertheless surprising. This was because its scarcity did not conform to the assumed wide usage of “ordinary dying” by palliative care clinicians and to the arguments that it has been utilised by medical professions for decades [27, 128, 129]. Given the lack of evidence for its use in peer-reviewed literature, it is difficult for us to comment on where this belief has come from or what it is based upon. For this reason, we urge caution regarding any proposed future use of “ordinary dying” in palliative care practice or policy, until a sound evidence base can be established and critical consideration is given to the appropriateness of its use to describe dying.

That it appears ‘ordinary’ dying is being widely used in palliative care without an established evidence base is as problematic as the uncritical use of ‘normal’, ‘natural’ and ‘typical’ dying. This is because there is evidence to show that what counts as ‘ordinary’ in wider healthcare settings is not a fixed category but, as Kaufman [130] has argued, a contested construct affected by a complex interplay of clinical and socio-cultural factors. For instance, interventions once regarded as ‘extraordinary’ (e.g. dialysis, organ transplants) can become routinised and perceived as ‘ordinary’ through institutional adoption, changes in socio-cultural expectations, and advances in medical practice. Understandings of what is ‘ordinary’ could then significantly affect issues from treatment decisions to resource allocation and medical futility debates [130–132]. This is sufficient to suggest that, if the concept of ‘ordinary’ dying follows a similar pattern as that of other practices or techniques that have become ‘ordinary’ in other areas of healthcare, something hitherto undefined and under-explored is being normalised as everyday end-of-life care. This is being done without a clear understanding of what that is or what the benefits, harms and limitations for patients or the healthcare system are. It is therefore imperative that research is undertaken to explore the values of ‘ordinary’ dying in palliative care, to better appreciate its likely impacts on enabling quality dying experiences and a ‘good’ death.

### Limitations

The findings of this review are a summary of all occurrences and usages, regardless of their frequency or salience. This descriptive nature without a more rigorous synthesis of recurring themes or patterns could consequently compromise the depth of this review. We did not account for a few grammatical variations of ‘ordinary’, ‘natural’ and ‘typical’ that may have consequently not been captured using our search terms which may have influenced the amount of existing empirical evidence on this matter. These variations include ‘ordinariness’ (for ‘ordinary’) and ‘typify’ (for ‘typical’). This could have influenced the depth of our findings. However, it would be unusual for a well-established understanding to exist in its grammatical variation (e.g. ‘ordinariness’ of dying) more than in its original form (e.g. ‘ordinary’ dying). In this regard, this limitation may implicitly show the lack of empirical studies on their understandings and hence highlight the need for further research. The limited descriptions of ‘normal’, ‘ordinary’, ‘natural’ and ‘typical’ dying in empirical palliative care literature published in English, also made any attempts at an in-depth synthesis somewhat futile.

But this could be precisely where our review could be deemed to have served its role as a scoping review: we have presented everything that has been published in peer-reviewed journals, without condensing and compromising any existing ‘knowns’ regarding these concepts. The limited empirical evidence also highlighted the paucity of empirical studies on what are held as definitive ideas for palliative care, thereby stressing the need for further studies on this matter to enable a more rigorous, evidence-based synthesis of these concepts.

## Conclusion

Our scoping review identified existing descriptions of ‘normal’, ‘ordinary’, ‘natural’ and ‘typical’ dying in empirical English-language palliative care literature. We found there was context-dependency and interchangeability in their understandings, highlighting their diversity and potential complexity in conceptualising them. Put simply, we found little that was normal, natural, ordinary or typical about ‘normal’, ‘natural’, ‘ordinary’ or ‘typical’ dying. Nonetheless, empirical evidence was limited and we could not determine with certainty whether the identified diversity reflected genuinely varied understandings or was a consequence of conceptual ambiguity, or – most likely – both. Importantly, this review found that understandings of ‘normal’, ‘ordinary’, ‘natural’ and ‘typical’ dying may play an important normative role in understanding and assessing the quality of dying experiences, as well as enabling good deaths in palliative care. However, the current paucity of empirical evidence limited our capacity to ascertain the precise benefits, harms and limitations of using ‘normal’, ‘ordinary’, ‘natural’ and ‘typical’ dying at this stage. We believe that with the growing interest and use of these terms among palliative care clinicians and policy makers, a significant programme of research is urgently needed to explore these central, yet chronically under-explored, concepts in palliative care.

## Supplementary Information

Supplementary Material 1. Supplementary Data 1.1. An example of search history (MEDLINE). It shows the search terms and filters used in this review. Supplementary Data 1.2 (Titled Supplementary Table 1). Data extraction table. It shows the types of data extracted from the included studies.

Supplementary Material 2. Supplementary Data 2 (Titled Supplementary Table 2). Summary of findings. It summarises the study characteristics of the included studies, and the findings relevant to this review.

## Data Availability

All data generated or analysed during this study are included in this published article. Search history and data extraction table have been provided in Additional file 1. Summary of findings on the explicit use of ‘normal’, ‘ordinary’, ‘natural’ and ‘typical’ dying has been provided in Additional file 2.
