# Supplementary material for: Dying as a ‘normal’, ‘ordinary’ or ‘natural’ process in palliative care: a systematic scoping review and narrative synthesis
Source: BMC Palliat Care. 2026 Jun 15;25:254. doi: 10.1186/s12904-026-02195-w (PMC13495523; doi:10.1186/s12904-026-02195-w)
Supplement: Supplementary file 1 [file 12904_2026_2195_MOESM1_ESM.docx]

Supplementary Document 1.1. An example of search history (MEDLINE)

Supplementary Document 1.2. Data extraction table

| Authors |  |
| --- | --- |
| Title |  |
| Publication year |  |
| Country | e.g. UK |
| Aim/s |  |
| Methods |  |
| Participants |  |
| Settings |  |
| Key study findings |  |
| Where was ‘normal’, ‘ordinary’, ‘natural’, ‘standard’, ‘typical’ or ‘usual’ explicitly used to describe dying? | e.g. Abstract, introduction |
| Terms used | e.g. normal |
| Who described dying as ‘normal’, ‘ordinary’, ‘natural’, ‘standard’, ‘typical’ or ‘usual’? | e.g. Authors, participants |
| What of dying was described as ‘normal’, ‘ordinary’, ‘natural’, ‘standard’, ‘typical’ or ‘usual’? | Direct quotes from the literature |
| Supplementary Table 1. Data extraction table | |
